# Editorial: Pathogenetic mechanism and therapeutic target for inflammation in autoimmune disease

**DOI:** 10.3389/fimmu.2024.1385936

**Published:** 2024-03-07

**Authors:** Jia Li, Minrui Liang, Hai-Feng Pan, Jian Gao, Liangjing Lu

**Affiliations:** ^1^ Department of Rheumatology, Ren Ji Hospital, Shanghai Jiao Tong University School of Medicine, Shanghai, China; ^2^ Department of Rheumatology, Huashan Hospital, Fudan University, Shanghai, China; ^3^ Institute of Rheumatology, Immunology and Allergy, Fudan University, Shanghai, China; ^4^ Huashan Rare Disease Center, Huashan Hospital, Fudan University, Shanghai, China; ^5^ Department of Epidemiology and Biostatistics, School of Public Health, Anhui Medical University, Hefei, Anhui, China; ^6^ Inflammation and Immune Mediated Diseases Laboratory of Anhui Province, Hefei, Anhui, China; ^7^ Shanghai Children’s Medical Center, School of Medicine, Shanghai Jiao Tong University, Shanghai, China

**Keywords:** pathogenetic mechanism, therapy, target, inflammation, autoimmune disease, biomarker

Autoimmune diseases encompass both autoimmune and autoinflammatory conditions, characterized by dysregulated immune responses targeting self-tissues and organs (1). Inflammation is a crucial driver of the pathogenesis of autoimmune diseases, leading to tissue damage and clinical manifestations. Notably, inflammation not only affects specific organs but can also cause systemic injury in various rheumatic disorders, including idiopathic inflammatory myopathies (IIM), inflammatory arthritis, inflammatory bowel disease (IBD), and systemic vasculitis (2–5). In certain instances, hyperinflammation, also known as cytokine storm, can occur, resulting in disastrous outcomes such as macrophage activation syndrome or secondary hemophagocytic lymphohistiocytosis (HLH) in active rheumatic diseases (6). Currently, therapeutic strategies targeting cytokines, signal pathways, or immune cells represent vital options in clinical practice (4, 7–9). Thus, the development of effective treatments for these debilitating conditions critically depends on understanding the underlying pathogenic mechanisms and identifying potential therapeutic interventions.

In this Research Topic, titled “*Pathogenetic Mechanisms and Therapeutic Targets for Inflammation in Autoimmune Diseases*,” we present a collection of 21 papers exploring various aspects of autoimmune disease inflammation, including genetic contributions, molecular mechanisms and pathogenesis, biomarkers, and therapeutic approaches.

Genetic factors significantly influence autoimmune disease susceptibility and outcomes. Huang et al.‘s analysis reveals genetic correlations and causal relationships between COVID-19 and osteoarthritis, suggesting a shared genetic predisposition and a non-causal impact of osteoarthritis on COVID-19 outcomes.

Several studies delve into the molecular mechanisms of underlying inflammation in autoimmune diseases. Xiang et al. provide a comprehensive summary of the classification, epidemiology, diagnosis, causative factors, pathogenesis, and targeted therapy related to inflammation in common autoimmune diseases, emphasizing the importance of maintaining a balance between autoimmune effects and immunomodulatory responses. Zhao et al. provides insights into the molecular pathogenesis of primary biliary cholangitis and explores the therapeutic benefits of natural products in managing this autoimmune liver disease [doi.org/10.3389/fimmu.2023. 1164202].Acquired bone marrow failure syndromes (BMFS),acquired aplastic anemia (AA) and poor graft function (PGF) after allogeneic stem cell transplantation (alloSCT),can lead to bleeding and infection due to persistent leukopenia and thrombocytopenia. Koldej et al. reported a shared inflammatory immunopathology between AA and PGF in primary patient samples, indicating a diminished state of immunoregulation and immunosurveillance. They proposed investigating novel treatments for acquired BMFS targeting the underlying immune dysregulation to restore and reset patient immunity, potentially leading to a cure for the condition.

Using single-cell RNA sequencing (scRNA-seq) data from peripheral blood mononuclear cells and histological staining, Zong et al. identify the high abundance of pro-inflammatory M1 macrophages and metabolic reprogramming of macrophages, highlighting potential targets for intervention. Similarly, Liu et al. report an increased level of CXCR5+TIM-3-PD-1+ stem cell-like T cells in chronic rhinosinusitis (CRS), suggesting that inducing CXCR5+TIM-3-PD-1+ T cell exhaustion could serve as an effective immunotherapy for CRS. Shao et al. elucidate the molecular mechanisms of pruritus in prurigo nodularis and propose potential therapeutic strategies based on their findings. Additionally, Fang et al. explore the role of DKK-1, a Wnt signaling antagonist, in the pathogenesis of ankylosing spondylitis through meta-analysis and Mendelian randomization, concluding no significant change in serum DKK-1 concentration between AS patients and healthy controls. Furthermore, Sandoval et al. demonstrate that activation of the aryl hydrocarbon receptor inhibits neuropilin-1 upregulation on IL-2-responding CD4+ T cells, highlighting a novel mechanism by which the aryl hydrocarbon receptor modulates effector CD4+ T cell responses.

Pattern recognition receptors (PRRs), such as toll-like receptors, are essential components of the innate immune system and are crucial for pathogen recognition in inflammation. Stierschneider and Wiesner provide an overview of the structure, function, and signaling pathways of TLR4, emphasizing its fundamental role in endothelial cells under physiological and inflammatory conditions, along with advances in TLR4 modulation strategies. Kulshrestha et al. assess the role of the alternative complement pathway in the pathogenesis of non-infectious uveitis. They measured complement components like C3b, factor B, and CFH as well as aqueous humor in patients with infectious and non-infectious uveitis as well as non-inflammatory controls, providing evidence implicating CFH and the activation of the alternative complement pathway in the pathophysiology of this condition.

A wide range of autoantibodies (Abs) are present in individuals with autoimmune diseases, exerting detrimental effects. Integral membrane proteins that are functionally active against G protein-coupled receptors (GPCRs) are most prevalent in SSc. Akbarzadeh et al. compile the effects of Abs directed against G protein-coupled receptors in systemic sclerosis (SSc), highlighting the pathophysiological role of these Abs and their potential implications for therapeutic development. Zhong et al. review the development of research on CH25H which is a protein to control lipid metabolism and immune cell function in intestinal immunity, aiming to identify early targets for the diagnosis and treatment of inflammatory bowel disease (IBD). Mangoni et al. investigate the role of the kynurenine pathway of tryptophan metabolism in the regulation of immune function and inflammation. They suggested significant alterations in significant alterations in tryptophan, kynurenine, 3-hydroxykynurenine, and quinolinic acid concentrations in rheumatic disease patients.

The association between alterations in gut microbiota and the etiology of human diseases has led to its emergence as a promising target for pharmacological modulation. Fan et al. review the advances in utilizing gut microbiota in enhancing the treatment efficacy of disease-modifying anti-rheumatic drugs in rheumatoid arthritis.

The authors also explore emerging biomarkers of autoimmune diseases. Huang et al. investigate differentially expressed genes (DEGs) between atherosclerosis (AS) and ulcerative colitis (UC), identifying protein tyrosine phosphatase, receptor type, C (PTPRC) as a key biomarker for the comorbidity of UC and AS, associated with dysregulated immunological and inflammatory responses. Li et al. identify metabolic markers of chronic obstructive pulmonary disease (COPD) using metabolomics and lipidomics in three different traditional Chinese medicine (TCM) patterns, highlighting potential targets for intervention. Research on specific biomarkers for lupus nephritis (LN) is ongoing. Yung and Chan review the correlation of components of the glycocalyx with disease activity in LN, suggesting their potential utility in diagnosis and prognostication.

The Research Topic also includes a study of novel treatment modality targeting inflammation and immune responses in autoimmune diseases. Zhao et al. assess the efficacy of Oridonin (ORI) in acute lung injury using Monoclonal antibody anti-CD31 antibody-conjugated nanoparticles (ACNPs) as a new nanodrug delivery system. They found that anti-CD31- ORI -NPs penetrated endothelial cell barriers and specifically accumulate in lung tissues at least three times compared to free ORI, offering a promising therapeutic strategy with high efficacy and low toxicity.

In addition, this Research Topic features two case reports. Yu et al. report a case of Kikuchi-Fujimoto disease, an inflammatory disorder related to viral infection, while Zhang et al. presents a case of systemic lupus erythematosus (SLE) and systemic sclerosis (SS) overlap syndrome presenting with epilepsy due to SRC-Hypertensive Encephalopathy-induced Posterior Reversible Encephalopathy Syndrome (PRES).

We are stepping into a new era in which we move beyond the traditional disease definition based solely on phenotype, instead incorporating the immunol phenotype and genotype, which might aid in treating diseases with greater precision and effectiveness. The papers in this Research Topic provide valuable insights into the complex interplay among the immune system, genetic factors, environmental triggers, and target tissues in the development and progression of autoimmune diseases. We hope that these findings will inspire further research and collaboration in the field of autoimmune disease inflammation, ultimately leading to more effective treatments for these challenging conditions.

## Author contributions

JL: Writing – original draft, Conceptualization. ML: Writing – review & editing. H-FP: Writing – review & editing. JG: Writing – review & editing. LL: Conceptualization, Writing – review & editing.
